# Self-Efficacy in Foot-Care and Effect of Training: A Single-Blinded Randomized Controlled Clinical Trial

**Published:** 2015-04

**Authors:** Alehe Seyyedrasooli, Kobra Parvan, Leila Valizadeh, Azad Rahmani, Maryam Zare, Tayyebeh Izadi

**Affiliations:** 1 Department of Medical and Surgical Nursing, School of Nursing and Midwifery, Tabriz University of Medical Sciences, Tabriz, Iran;; 2 Department of Pediatric Nursing, School of Nursing and Midwifery, Tabriz University of Medical Sciences, Tabriz, Iran;; 3 Department of Community Health Nursing, School of Nursing and Midwifery, Shiraz University of Medical Sciences, Shiraz, Iran

**Keywords:** Diabetes Mellitus, Diabetic Foot, Education, Group Training

## Abstract

**Background:**

Diabetes mellitus (DM) is one of the most common metabolic and non-communicable disorders worldwide and the mortality rates caused by the complications associated with the disease, such as diabetic foot ulcer, is increasing dramatically. Patient education is considered as an essential part of controlling DM. Therefore, we aimed to compare the effects of individual and group training methods on self-efficacy in foot care among the patients with DM.

**Methods:**

In this single-blinded, randomized controlled clinical trial, we enrolled 150 patients with type 1 and 2 DM. The final participants were randomly assigned into two intervention groups (collective and individual training group) and a control group. Data were collected using foot-care self-efficacy questionnaire (Corrbet, 2003). A research assistant collected the data by interviewing the participants using the questionnaire once before and once one month after the intervention. The participants of the intervention groups attended a training program consisting of three sessions per week for one week. Statistical descriptive tests such as mean and standard deviation (SD) percentage were used to describe the features of the data inferential statistics test such as Chi-square, independent t-test and repeated measures analysis of variance and analysis co-variance (ANOVA, ANCOVA) tests were also used as appropriate. The significance level was set at <0.05.

**Results:**

The results indicated that there was no significant difference between the three groups regarding the mean of self-efficacy scores before foot-care training intervention (P=0.39). But, comparison of the scores before and after the intervention showed that both group and individual training interventions increased the patients’ self-efficacy (P≤0/05).

**Conclusion:**

It can be concluded that both group and individual training approaches could increase foot care self-efficacy in the patients with DM.

**Trial Registration Number: **IRCT201203086918N6.

## Introduction

Diabetes Mellitus (DM) is one of the most important and common non-communicable metabolic disease worldwide which is also known as a major contemporary public health issue. DM is characterized by glucose intolerance resulting from imbalance between insulin supply and metabolic demand. The disease is caused by defects in insulin secretion or inefficiency of secreted insulin due to high blood glucose levels.^1^^,^^2^

According to the statistics published by Iranian Diabetes Association, currently 7 million people (9.8%) are suffering from the mentioned disease.^3^ According to the International Diabetes Federation, in the case of failure in prevention of this epidemic, the number of people with diabetes will increase from 366 million people in 2011 to 522 million in 2030.^3^

Diabetic foot complications are a common global problem as there is no region in the world where no report is given on the development of such problems.^4^ Based on World Health Organization criteria, the most common and serious diabetic food complications are ulceration, infection, destruction of deep tissues associated with neurological abnormalities and various degrees of peripheral vascular disease in the lower limb.^4^^,^^5^

According to epidemiological studies, 2.5% of the people with diabetes mellitus (DM) develop foot ulcers annually and approximately 15% of them will develop a diabetic foot ulcer at least one time during their course of disease.^6^^,^^7^ Research has shown that managing foot ulcers for reducing the risk of amputation has a significant effect on the patients’ performance due to some reasons. Firstly, the patients play an effective role in their own treatment. Secondly, a 24-hour monitoring of the patients by their physicians seems impossible. Besides, the results of other studies suggest that it is possible to prevent 85% of lower extremity amputations by implementing diabetes educational programs with special emphasis on foot care education.^8^^,^^9^

According to previous studies, applying educational strategies is the most important method of preventing diabetic foot ulcers.^10^^,^^11^ The patients will participate in their treatment decisions more effectively if they can obtain sufficient knowledge about their disease through education.^1^ Patient education program not only decreases anxiety, but also helps to increase satisfaction, independence and participation in self-care programs. Besides, reduced length of hospital stay and complications associated with the disease as well as increased longevity and health promotion are other achievements of such program.^12^ Therefore, the patients with DM should be trained to take care of their feet by cutting nails carefully and properly, daily inspection of their feet, using suitable footwear, keeping feet dry, and visiting a podiatrist in case of any changes.^13^^,^^14^

Self-efficacy is one of the basic and fundamental concepts of a social cognitive theory which was initially developed by Albert Bandura. It is defined as one’s belief in one’s own ability to perform specific tasks successfully and expect their outcomes. Bandura believes that individuals’ self-efficacy and capabilities can be increased if they are provided with a proper context in which they can acquire required skills and knowledge. Self-efficacy can also affect people’s motivation and make them exert greater effort and persist longer in the behaviors. Moreover, it plays an important role in treatment of chronic diseases.^15^

So far, no significant difference has been observed about the effects of different educational methods in this regard. Very few studies can be found in the literature addressing directly the comparison of individual and group education in foot care self-efficacy behavior among the patients with Diabetes Mellitus. On the other hand, individual and group training approaches are commonly used for routine care. Accordingly, their relative effects cannot be clearly indicated. 

Conducting the present study seemed necessary due to the following reasons: the importance of preventing DM complications, lack of reports on suitable educational methods for such patients, and importance of the disease in Iran. Hence, we aimed to compare the effects of individual and group training methods on foot-care self-efficacy in patients with DM. 

## Patients and Methods

**Figure 1:**
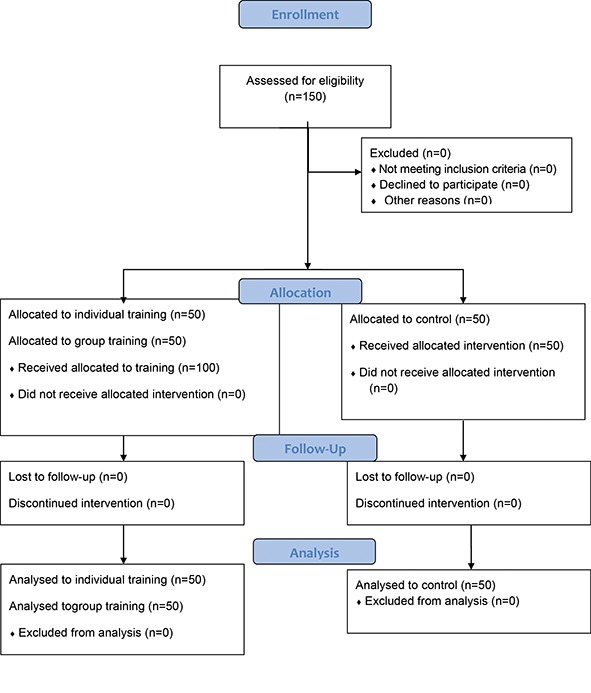
Consort flow diagram of the participants

This study was approved by the Ethics Committee of Tabriz University of Medical Sciences. In this single-blinded, randomized controlled clinical trial which was done during September 2012-March 2013, the sample size was calculated using the formula of Estimation of Mean sample size for comparative studies in accordance with a pilot study with a sample size of 20 patients in each group; the mean and standard deviation were respectively 18.9±5.6, 20.9±6.1, 17.8±4.9, d= 3.1, S1=6.1, S2=4.9, , β=0.2. The sample size was calculated as 50 in each group. A simple random sampling method was used through the table of random numbers to randomize the participants into intervention and control groups. The patients were selected using a simple sampling method. We enrolled 150 patients aged 18-65 years with type 1 and 2 DM. The final participants, who met inclusion criteria, were randomly assigned into two intervention groups (collective training group [n=51], individual training group [n=49]) and a control group [n=50] (Figure 1). The participants were selected from the population who referred to Nader Kazemi health center which is the main diabetic center in Shiraz and affiliated to Shiraz University of Medical Sciences, Shiraz, and southwest Iran.

Inclusion criteria were age of 18 years or older, a confirmed diagnosis of DM (type 1 or 2) by an endocrinologist, the participant’s awareness of the diagnosis, physical and mental ability to attend all training sessions, foot self-care ability, no history of education in medical-related fields or taking any courses in foot care and lack of gestational DM. However, exclusion criteria were failure to complete the training program, receiving any training in the related fields during the study and having a diabetic foot ulcer.

After obtaining permission from the authorities of the mentioned clinic, we selected our participants who had inclusion criteria from the population of the patients with DM who referred to the clinic. The aim and method of the research were explained to them during a telephone conversation and written informed consent was taken from the patients who were willing to participate in the study. 

Data were collected using the foot-care self-efficacy questionnaire (Corbett 2003). The scale consists of 7 items presented on the 5-point Likert Scale ranging from 0 “Strongly Disagree” to 5 “Strongly agree”. Scores are calculated as follows: for items 1, 4, 5, and 7: Strongly agree=5 and for items 2, 3and 6: Strongly disagree=5.^13^

To collect the data, foot care self-efficacy questionnaire was used and translated. In this way, two English language experts translated the questionnaire into Persian separately. Then, by comparing the two translated versions, a Persian version was prepared. In this step, the objective was to ensure that there is no ambiguity in the questionnaire’s questions and when a different person reads it, she/he can reach a common understanding of the questions. In the next stage, back-translation (Persian into English translations) was performed by an English language expert who was unaware of the content of the original questionnaire. In the final step, by comparing the original and translated versions, Persian translation was applied. Its reliability was also confirmed by conducting a pilot study on a similar group (n=25) and Cronbach’s alpha was calculated as 0.84.

A research assistant collected the data by interviewing the participants using the questionnaire before the beginning of the study. Afterward, the researcher began instructing the patients of the intervention groups. The participants of the collective training group participated in the program in groups of 3 to 5 patients. The patients in both groups attended a training program consisting of three sessions per week for one week. A post-test was taken again by the research assistant from the participants of both groups one month after the end of the intervention. The participants in the control group only received routine instructions of the outpatient ward without undergoing any intervention. After the training period, the control group received the educational package. The aim, length and time of the program were the same for both intervention groups (Figure 1).

The intervention and control groups were matched for age, sex and educational status. The collected data were analyzed using SPSS software, version 19. Statistical descriptive tests such as mean and standard deviation (SD) percentage were used to describe the features of the data and inferential statistics test such as Chi-square, independent t-test and repeated measures analysis of variance and covariance (ANOVA, ANCOVA) tests were also used as appropriate. The significance level was set at <0.05. 

## Results

The mean±SD age of the participants in the individual training, collective training and control groups were 46.9±17.6, 47.4±16.7, and 40.6±16.3, respectively. The independent t-test showed no significant difference between the groups in this regard (P=0.08). Socio-demographic characteristics of the participants are shown in Table 1. The results of Chi-square and paired t-test indicated that the participants of the three groups were homogenous in terms of their age, sex, type of DM, marital status, educational level, occupational status, the type of treatment, duration of the disease.

**Table 1:** Frequency distribution of socio-demographic characteristics of the participants with DM

**Variable**	**Subcategories **	**Total N(%)**	**P value**
Types of DM	Type 1	50 (33.3)	0.023
	Type 2	100 (66.7)	
Sex	Male	74 (49.3)	0.918
	Female	76 (50.7)	
Marital Status	Single	35 (23.3)	0.66
	Married	95 (63.3)	
	Widow	15 (10)	
	Divorced	5 (3.3)	
Educational Level	Primary Education	21 (14)	0.083
	Secondary Education	30 (20)	
	High School Diploma	60 (40)	
	Higher Education	39 (26)	
Occupational Status	Unemployed or Retired	50 (33.3)	0.531
	Housewife	51 (34)	
	Self-employed	28 (18.7)	
	Clerk	20 (13.3)	
	Laborer	1 (0.7)	
Type of Treatment	Insulin Therapy	71 (47.3)	0.154
	Pill-Treatment	93 (52.7)	0.72
Glycemic Control	Yes	137 (91.3)	0.564
	No	13 (8.7)	

Most of the participants (50.7%) were women. 63.3% of all the patients were married. The majority of the participants had high school diploma (40%) and type 2 DM (66.7%). 62% of them were treated with diabetes pills and 30% had a history of DM for 1 to 5 years. Because in this study the blood sugar level of the patients was not important, the patients were asked whether they monitor their blood glucose or not. Finally, 91% of the patients were regularly monitoring their blood glucose (Table 1).

Furthermore, the results of ANOVA test in Table 2 demonstrated that there was no significant difference among the three groups regarding the mean of self-efficacy scores before foot-care training intervention (P=0.39). Moreover, the results demonstrated that there was a significant difference among the three groups regarding the mean of self-efficacy scores after foot-care training. Between groups comparison of the mean of self-efficacy scores was performed using paired t-test. The mean of self-efficacy score increased 10.1 in the individual training group; the results of paired t-test showed a significant difference between them (P<0.005). Also, the mean of self-efficacy score increased 9.3 in the group training group, showing a significant difference between them (P<0.005). The mean of self- efficacy score increased 0.4 in the group training and the paired t-test showed no significant difference between them (P=0.07). (Table2)

**Table 2:** Comparison of foot care self-efficacy in the participants of the three groups before and after the intervention

**Groups**	**Before ** **mean±SD**	**After ** **mean±SD**	**P value (paired t-test)**
Individual Training Group	18.6±6.22	28.7±6.06	0.001
Collective Training Group	18.9±6.1	28.2±5.07	0.001
Control Group	17.4±5.5	17.8±4.9	0.07
P value (ANOVA)	0.39	0.001	

Considering the fact that self-efficacy scores among the three groups before the intervention was different, to control the possible confounding effect of these factors, the results were analyzed using analysis of covariance. The results of ANCOVA showed that by controlling the mean of self-efficacy before and after the intervention, there were still significant differences between the three groups (Table 3).

**Table 3:** Comparison of the mean change scores of self-efficacy in the three groups

**Group**		**Mean Difference±SD**	**P value**
Individual Training Group	Group Training	0.46±1.07	0.9
	Control Group	10.9±1.08	0.001
Group Training Group	Individual Training Group	-0.46±1.07	0.9
	Control Group	10.5±1.07	0.001
Control Group	Individual Training Group	-10.9±0.84	0.001
	Group Training Group	-10.5±0.83	0.001

## Discussion

In extensive review of the literature in the years 2003-2014 by the researchers in relation to the variables of self-efficacy, individual and group training, foot care and diabetes, we could find no study. Therefore, the findings of the study indicated the effect of education on diabetes self-efficacy.

In another study, there was an attempt to “determine the effectiveness of the diabetes educational program on glycemic control, self-management and the self-efficacy in patients with type 2 diabetes”. The results indicated an increase in the self-efficacy of patients after training, but this increase was not statistically significant, (P>0.05).^16^

A single-blind clinical trial determined “the effect of education on knowledge, self-management behaviors and self-efficacy in patients with type 2 diabetes”; the patients were divided into groups of 12-7 in two 45-minute training session. Results showed that the self-efficacy of the patients in the intervention group increased and this increase was statistically significant (P<0.05).^17^

The results of another study in the field of education of foot care and reduction of the risk of foot ulcers showed increased patient self-efficacy.^18^

There is no doubt that the above-mentioned studies are in line with the current study and confirm its results. But due to the fact that the scope of this study was to examine both individual and group training, and according to tables 2 and 3, foot care education in both teaching methods (individual and group) increased the self-efficacy in the foot care in patients with type 1 and 2 diabetes, neither of the methods is preferred over the other one and the use of the both methods has the same effect on increasing the patients’ self-efficacy. These two methods can be used together in line with the complete foot care processes. A number of studies have focused on the field of individual and group training.

The results of a study on individual and group training combined in diabetic patients revealed an increase in this category of patients’ self- care; this supports the findings of this study.^19^

Results of the study reflect the fact that training has been effective in diabetics group but they suggested that group training sessions with individual components of the evaluation standard  can be a safe and effective method for caring diabetic patients.^20^ Moreover, some study results indicated that individual training in patients with newly diagnosed diabetes led to a better control of blood sugar in the trained group.^21^

The findings of another study also showed that the impact of individual and group training methods was the same.^22^ In this regard, the results of study indicated the fact that the impact of individual and group education on self-care practices of people with diabetic foot ulcers was not statistically significant.^12^

In a meta-analysis, the emphasis was on group training.^23^ Also, the conclusion of other studies implied that individual and group training in diabetes is equally effective.^24^ Results of another study showed that although the impact of the group training was more than individual training, there were no significant differences between both methods and the diet and control of hypoglycemia have been improved.^25^

Other researchers showed that both methods can be effective in diabetes while the impact of blood glucose control in group training has more efficacy than individual training.^26^ Also, researchers found that face to face education is effective and it is a practical method to enhance the knowledge and performance of foot care in diabetic patients.^27^

Moreover, the results of another study showed that although the outcome of the effectiveness of individual and group training is the same, the evidence suggests cost effectiveness, better acceptance, changes in lifestyle, and higher level of satisfaction with treatment after group training.^28^

One of the limitations of the present study is that our results can only be generalized to those patients who meet the inclusion criteria. Generalizability of the obtained results requires repetition of the study on a different study population. Furthermore, another limitation, which was out of the researcher’s control was mental and physical condition of the studied population at the time of completing the questionnaire that could affect the participants’ responses to questions.

Therefore, nursing managers are recommended to establish patient education units in diabetes clinics and implement codified group training programs so that they can reduce the patient’s problems and hospital costs associated with the disease. 

## Conclusion

Our findings showed that both group and individual training approaches could increase foot care self-efficacy in the patients with DM. Both types of training can play a positive role in prevention of diabetic foot. It seems that both educational methods together can be effective, while group-based training approach is more economical in terms of time and cost than individual-based training approach. The patients can learn certain behaviors in public which cannot be learnt through individual training. Moreover, in a group training approach, patients feel more secure, make use of each other’s experience, and support each other. Consequently, their internal and external motivations help them gain positive insights and stabilize the learned concepts. Another priority of group training over individual training program is that the educators train more patients in a shorter period of time. 
